# Editorial: Superwetting Interfaces for Oil/Water Separation

**DOI:** 10.3389/fchem.2021.667106

**Published:** 2021-03-24

**Authors:** Jiuyang Lin, Qin Chen, Moyuan Cao, Jingwei Hou, Wenyuan Ye

**Affiliations:** ^1^Fujian Provincial Engineering Research Center of Rural Waste Recycling Technology, School of Environment and Resources, Fuzhou University, Fuzhou, China; ^2^Key Laboratory for Green Chemical Technology of Ministry of Education, School of Chemical Engineering and Technology, Tianjin University, Tianjin, China; ^3^School of Chemical Engineering, University of Queensland, St. Lucia, QLD, Australia; ^4^Fujian Provincial Key Laboratory of Soil Environmental Health and Regulation, College of Resources and Environment, Fujian Agriculture and Forestry University, Fuzhou, China

**Keywords:** oil spill, industrial oily wastewater, superhydrophilicity, superhydrophobicity, oil/water separation

Ever-increasing instances of oil spill accidents and the disposal of urban sewage and industrial oily wastewater have become a global challenge that poses a serious threat to the ecosystem (Zhang et al., 2018; Zhao C. et al., 2021). The effective separation of oil and water from these waste streams has drawn increasing attention under the advanced framework of sustainability for wastewater treatment (Lin et al., 2020; Liu et al., 2021). However, conventional methods, including coagulation, centrifuging, sedimentation, air flotation, biological degradation, and membrane technologies, fail to effectively separate oil and water, especially in oil/water emulsion solutions (Yu et al., 2017; Lee et al., 2020; Zhao Y. et al., 2021), compromising the sustainable management of oily wastewaters. Therefore, there is a strong need to develop superwetting interfaces (e.g., superhydrophobic or superhydrophilic interfaces) for effective oil/water separation.

Generally, two prevalent strategies, i.e., reduction in surface energy and surface decoration via nano/micro-structure, are proposed in the design of superhydrophobic interfaces for removing oil in oil/water separation (Zhang et al., 2013; Dalawai et al., 2020). To achieve this goal, great efforts were made to tailor the interface of these superwetting materials. Zhang et al. synthesized alkyl chain grafted-reduced graphene oxide for constructing the superhydrophilic membrane by vacuum filtration. The fabricated membrane showed an acceptable separation efficacy in an n-propanol/water mixture. Khan et al. employed the method of ultrashort femtosecond laser pulses to nanostructures the stainless steel and copper meshes with superhydrophobicity, which yielded a separation efficiency of 98% for an oil/water mixture. Zulfiqar et al. summarized the methods of superhydrophobic interface engineering based on various nanomaterials, such as silica-based and carbon-based nanomaterials. The introduction of these silica-based (e.g., saline-based) and carbon-based nanomaterials onto the material surfaces could enhance the surface roughness with reduced surface energy for effective oil/water separation.

The interfaces with superhydrophilicity are underwater superoleophobic, which would remove water but retain oil. These superhydrophilic interfaces are an important alternative for effective oil/water separation (Shi et al., 2016; Lv et al., 2020). The key challenge to construct the superhydrophilic interfaces is to introduce the hydrophilic group to the interfaces via physical, chemical, or physico-chemical strategies (Zarghami et al., 2019). Bian et al. employed low-cost materials, i.e., filter paper and zeolite layer, which commonly exist in our life or nature for oil/water separation. Due to the inherent microscale porous structure, micro/nanoscale hierarchical rough surface texture, and hydrophilic surface properties, these two materials showed strong water-absorption ability, resulting in superhydrophilicity and underwater superoleophobicity without further treatment. Furthermore, the separation efficiency in an oil/water mixture can reach >96.0%, showing great potential in oil/water separation. Zulfiqar et al. also summarized the metal oxide nanoparticles, including iron oxide, titanium oxide, and zinc oxide, to design the superhydrophilic interfaces. This review paper concludes that iron-based oxides can be more competitive, providing the unique advantage of reusability and fast separation due to their magnetic properties. Additionally, Feng and Yong summarized the strategy of femtosecond laser processing for the fabrication of superhydrophilic interfaces. This review paper concludes that femtosecond laser processing can construct superwetting microstructures on diverse materials or membranes by a single-step ablation. However, several challenges should be addressed to satisfy its large-scale practical application.

This Research Topic discusses the latest innovations in the preparation and application of “oil-removing” type materials with superhydrophobicity and superoleophilicity (that selectively filter or absorb oil from oil/water mixtures), “water-removing” type materials with superhydrophilicity and superoleophobicity (that selectively separate water from oil/water mixtures). In the future, superwetting materials will provide a feasible solution in addressing global oil spill accidents and realizing resource recovery from oily industrial wastewaters. The cost-effective strategy of constructing superwetting interfaces with strong durability/stability in extreme environments and the design of practical large separation instruments are two key research directions that should be paid more attention to in the future. Moreover, the separation of high-viscosity crude oils and surfactant stabilized emulsions needs to be considered as well in future research.

## Author Contributions

JL, QC, MC, JH, and WY contributed to the writing of this editorial. All the authors contributed to the article and approved the submitted version.

## Conflict of Interest

The authors declare that the research was conducted in the absence of any commercial or financial relationships that could be construed as a potential conflict of interest.
